# Comparison of Diagnostic Accuracy and Diagnostic Adequacy Between Endoscopic Ultrasound-Guided and Percutaneous Liver Biopsies: A Meta-Analysis of Randomized Controlled Trials and Observational Studies

**DOI:** 10.7759/cureus.59636

**Published:** 2024-05-04

**Authors:** Mansoor Ahmad, Taslova Tahsin Abedin, Faria Khilji, Kinan Obeidat, Lam Vinh Sieu, Sandipkumar S Chaudhari, Divine Besong Arrey Agbor, Danish Allahwala

**Affiliations:** 1 Medicine, Rehman Medical Institute, Peshawar, PAK; 2 Medicine, Sylhet MAG Osmani Medical College, Sylhet, BGD; 3 Internal Medicine, Tehsil Headquarter Hospital, Shakargarh, PAK; 4 Internal Medicine, Quaid-e-Azam Medical College, Bahawalpur, PAK; 5 Internal Medicine, University of Texas Medical Branch at Galveston, Galveston, USA; 6 Medicine, Moscow State University of Medicine and Dentistry, Moscow, RUS; 7 Cardiothoracic Surgery, University of Alabama at Birmingham, Birmingham, USA; 8 Family Medicine, University of North Dakota School of Medicine and Health Sciences, Fargo, USA; 9 Clinical Research and Internal Medicine, California Institute of Behavioral Neurosciences & Psychology, Fairfield, USA; 10 Internal Medicine, Richmond University Medical Center, New York City, USA; 11 Nephrology, Fatima Memorial Hospital, Karachi, PAK

**Keywords:** meta-analysis and systematic review, adequacy, accuracy, percutaneous liver biopsy, eus-guided liver biopsy

## Abstract

A liver biopsy (LB) is a crucial diagnostic tool for evaluating liver diseases and is traditionally performed percutaneously under ultrasound guidance (PC-LB). However, endoscopic ultrasound-guided liver biopsy (EUS-LB) has emerged as an alternative approach, offering potential advantages over conventional techniques. This systematic review and meta-analysis aimed to compare the effectiveness and safety of EUS-LB using modern core biopsy needles with PC-LB.

A comprehensive literature search identified nine studies involving 785 patients that met the inclusion criteria. The meta-analysis evaluated three primary endpoints: diagnostic adequacy, diagnostic accuracy, and adverse event rates. The results indicated no significant difference in overall diagnostic adequacy (odds ratio: 0.446, 95% CI: 0.192-1.031) or diagnostic accuracy (odds ratio: 1.646, 95% CI: 0.224-12.09) between EUS-LB and PC-LB. Furthermore, the combined occurrence of adverse events did not differ significantly between the two procedures (odds ratio: 0.653, 95% CI: 0.298-1.431). However, PC-LB demonstrated superiority in obtaining a higher number of complete portal tracts (mean difference: -0.985, 95% CI: -1.753 to -0.218), indicating better specimen quality.

While both EUS-LB and PC-LB exhibited similar diagnostic performance and safety profiles, PC-LB provided higher-quality specimens, which may be advantageous in cases where accurate diagnosis and staging are critical, such as the evaluation of liver fibrosis. Clinicians should consider factors like specimen quality, procedural preferences, and local expertise when selecting the appropriate biopsy approach tailored to individual patient needs and clinical circumstances.

## Introduction and background

A liver biopsy (LB) serves as a crucial diagnostic tool for identifying both focal liver lesions and diffuse liver disorders. Traditionally, biopsy samples are obtained percutaneously under ultrasound guidance [1]. However, in certain clinical scenarios, such as coagulopathy and ascites, where percutaneous sampling cannot be done, alternative approaches such as transjugular liver biopsy (TJ-LB) or plugged LB are employed [2-3]. With the expanding utilization of endoscopic ultrasonography (EUS) in clinical settings, EUS has emerged as a viable method for liver tissue sampling, offering distinct advantages over conventional biopsy techniques. Endoscopic ultrasonography offers improved accessibility, real-time visualization, enhanced safety, detection of smaller lesions, concurrent interventions, outpatient procedure feasibility, and avoidance of ascites for liver tissue sampling compared to conventional biopsy techniques [3]. A recent analysis highlighted that percutaneous liver biopsy (PC-LB) carries a low risk of major complications, including death, moderate-to-severe pain, and major bleeding [4]. Transjugular liver biopsy is particularly favored in patients at greater risk, including those with coagulopathy, substantial ascites volume, or coagulation disorders, as well as individuals deemed clinically unstable to undergo percutaneous procedures [5]. 

Since its initial description in the year 2007, EUS-LB has gained traction as an appealing method for procuring LB specimens to diagnose and stage chronic hepatic diseases [6]. Endoscopic ultrasonography enables access to both liver lobes, and the fanning technique facilitates the retrieval of multiple cores and representative samples. Patients experience minimal discomfort because they are sedated during the procedure. Additionally, EUS assists in detecting smaller liver lesions and contributes to the assessment of surrounding abdominal structures [7]. 

A meta-analysis indicated a histologic diagnosis rate of 93.9%, along with an adverse event incidence of 2.3% in connection with EUS-LB [6]. As compared to PC-LB, EUS-LB yielded similar outcomes regarding complete portal tracts (CPT) and total specimen length (TSL) obtained, with no notable disparity in the occurrence of adverse events [8]. In recent times, interest has been growing in EUS-LB, especially with the emergence of newer or second-generation needle designs. These advanced designs offer improved performance for tissue acquisition when compared to conventional options like the 19G TruCut needle [9]. Considering the recent studies conducted to compare EUS-LB with second-generation needles and PC-LB, we undertook this updated systematic review and meta-analysis to compare the effectiveness and safety of EUS-LB with second-generation needles and PC-LB. 

## Review

Methodology

Search Strategy

Two reviewers (FK and KK) independently conducted a thorough search of relevant medical literature to identify studies discussing the outcomes of two procedures: EUS-LB using modern core biopsy needles and PC-LB. The search was performed on April 10, 2024, using databases such as PubMed, Web of Science, the Excerpta Medica database (EMBASE), and the Cochrane Library. Keywords used to search for articles included EUS, percutaneous, and liver biopsy, along with their synonyms and Medical Subject Headings (MeSH) terms. The search encompassed studies published in the English language. Additionally, we screened the reference lists of all eligible records.

Study Selection

This comprehensive review focused on studies that compared EUS-LB with PC-LB. It included a range of study types, such as randomized controlled trials (RCTs), case-control studies, and cohort studies, that evaluated the outcomes of both procedures. All studies were considered regardless of their location, sample size, research environment, or follow-up duration, as long as the data for analysis were available. Studies without a comparison group were excluded, along with case reports, case series studies, reviews, meta-analyses, and editorials. Two authors independently reviewed all records obtained from database searching. After duplicate removal, screening was performed using pre-defined inclusion and exclusion criteria. A detailed assessment was done of all eligible articles to assess whether they could be included in the meta-analysis or not.

Data Abstraction and Outcomes

Two investigators meticulously assessed each study and gathered data on significant findings using a predetermined data collection template developed in Microsoft Excel (Microsoft Corp., Redmond, WA). This meta-analysis concentrated on three primary endpoints: the quality of tissue samples (diagnostic adequacy), the precision of diagnoses (diagnostic accuracy), and the occurrence of any adverse events. A satisfactory sample denoted its capability to furnish adequate information for diagnosing the disease and determining its severity, irrespective of sample dimensions. Accuracy denotes the percentage of patients accurately diagnosed, accounting for both positive and negative outcomes. 

Statistical Analysis

We used the Comprehensive Meta-Analysis software, version 4 (Biostat, Englewood, NJ) for all analyses. To compare categorical outcomes between the two study groups, we calculated odds CPTratios with 95% confidence intervals, and for continuous variables, we calculated mean differences (along with a 95% CI for variables. Statistical significance was considered when the p-value was below 0.05. To address differences in study results and outcomes, we applied a random effects model to calculate combined estimates. Heterogeneity was computed using I-square and Cochran's Q statistics. An I-square value of 50% or more shows significant heterogeneity. 

Results

**Figure 1:**
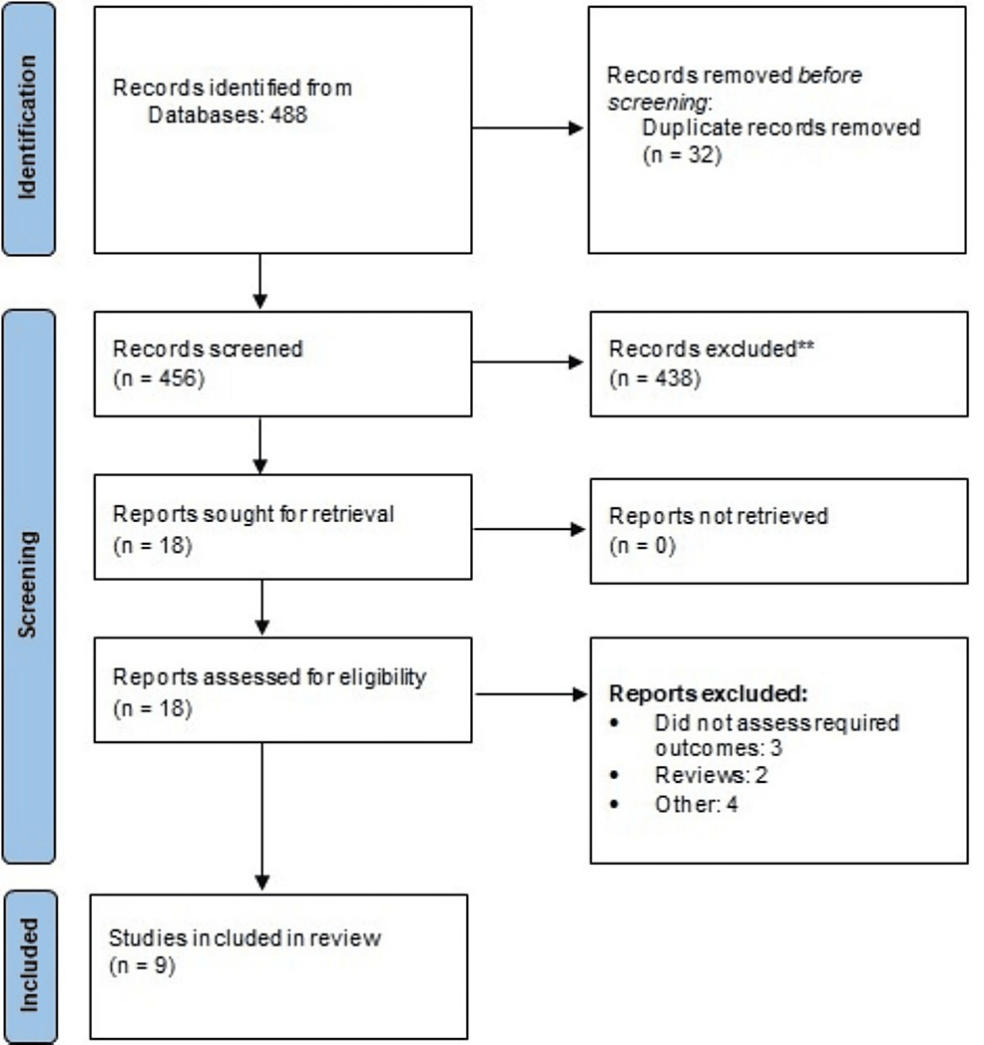
A PRISMA flowchart showcasing the study selection process PRISMA: Preferred Reporting Items for Systematic Reviews and Meta-Analyses

Online database searching yielded 488 records. Upon screening of articles after removing duplicates, 18 studies passed through initial screening. Finally, nine studies were included in this meta-analysis, comprising 785 patients. Figure 1 shows the process of study selection. Table 1 shows the characteristics of the included studies. Out of nine studies, six were observational and three were RCTs.

**Table 1:** Characteristics of the included studies EUS-LB: endoscopic ultrasound-guided liver biopsy; PC-LB: percutaneous liver biopsy; RCT: randomized controlled trial

Study ID	Design	Region	Groups	Sample size
Ali et al., 2020 [10]	Observational	United States	EUS-LB	30
			PC-LB	60
Ali et al., 2023 [11]	RCT	United States	EUS-LB	40
			PC-LB	40
Bang et al., 2021 [12]	RCT	United States	EUS-LB	21
			PC-LB	19
Bhogal et al., 2020 [13]	Observational	United States	EUS-LB	135
			PC-LB	287
Facciorusso et al., 2021 [14]	Observational	Italy	EUS-LB	54
			PC-LB	62
Farrel et al., 2023 [15]	Observational	United States	EUS-LB	30
			PC-LB	60
Nallapeta et al., 2023 [16]	RCT	United States	EUS-LB	36
			PC-LB	44
Patel et al., 2022 [17]	Observational	United States	EUS-LB	53
			PC-LB	20
Yewale et al., 2023 [18]	Observational	India	EUS-LB	19
			PC-LB	18

Meta-analysis of Outcomes

**Figure 2:**
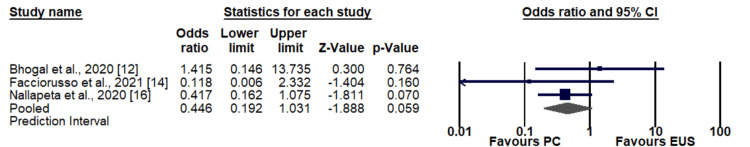
Comparison of diagnostic adequacy between two groups Sources: [13-14, 16]

Diagnostic adequacy: Three studies compared the diagnostic adequacy of two procedures, PC-LB and EUS-LB. The results, as depicted in Figure 2, revealed that there was no statistically significant difference in the overall diagnostic adequacy between the two procedures. The odds ratio of 0.446 with a 95% confidence interval of 0.192-1.031 suggests that the diagnostic adequacy was comparable between the two methods. Additionally, the study results did not exhibit significant heterogeneity, as indicated by the I-square value of 0% and a p-value of 0.411, suggesting consistency among the individual studies included in the analysis.

**Figure 3:**
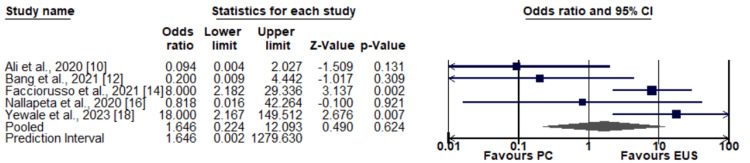
Comparison of diagnostic accuracy between two groups Sources: [10, 12, 14, 16, 18]

Diagnostic accuracy: In Figure 3, a comparative assessment of diagnostic adequacy between PC-LB and EUS-LB is depicted. The results indicated that there was no significant variance in the overall diagnostic accuracy between the two techniques (odds ratio: 1.646, with a 95% confidence interval ranging from 0.224-12.09), as depicted in Figure 3. Significant heterogeneity was reported among the study results (I-square: 69%, p-value: 0.011).

**Figure 4:**
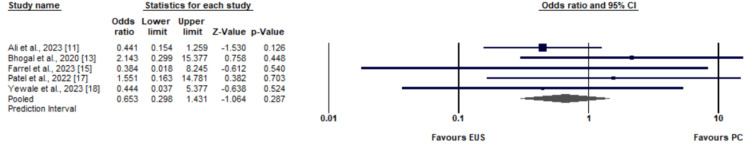
Comparison of adverse events between two groups Sources: [11, 13, 15, 17-18]

Adverse events: The analysis did not reveal a significant difference in the overall occurrence of adverse events between the PC-LB and EUS-LB procedures, as shown in Figure 4. The same figure illustrates that the risk of adverse events was comparable between the two groups, with an odds ratio of 0.653 and a 95% confidence interval of 0.298-1.431, indicating no statistically significant disparity. Furthermore, the study results exhibited consistency, as evidenced by the lack of significant heterogeneity among the included studies, with an I-square value of 0%.

**Figure 5:**
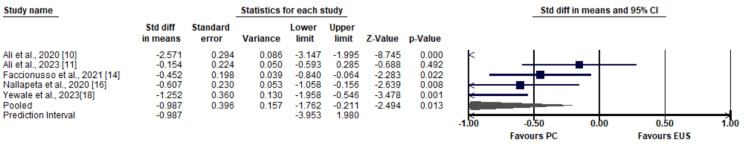
Comparison of mean CPTs between two groups CPT: complete portal tract Sources: [10-11, 14, 16, 18]

Complete portal tract: Five studies evaluated and compared the average number of CPTs obtained from EUS-LB and PC-LB procedures. As illustrated in Figure 5, the mean number of CPTs was significantly higher in patients who underwent PC-LB compared to those who underwent EUS-LB (mean difference: -0.987, 95% confidence interval: -1.762 to -0.211). However, the study results exhibited substantial heterogeneity among the included studies, as indicated by the high I-square value of 92.02%.

Discussion

This meta-analysis, based on nine studies encompassing both observational studies and RCTs, indicates that both PC-LB and EUS-LB demonstrate similar adequacy, with no significant differences observed between the two in terms of adverse events. These findings align with the meta-analysis performed by Chandan et al., which similarly concluded that the combined diagnostic adequacy and risk of adverse events did not notably differ between the two groups. However, PC-LB displayed superiority in relation to the average number of CPT [19]. 

As advancements in endohepatology continue, driven by the emergence of advanced EUS-guided biopsy needles, there is an increasing amount of evidence suggesting that EUS-LB is considered to have fewer contraindications compared to the conventional PC-LB method [20]. An EUS-LB offers several advantages, including the capability to perform multiple needle passes following a single liver capsule puncture, simultaneous assessment and treatment of luminal pathology, and quicker recovery compared to alternative methods. However, potential drawbacks of EUS-LB include increased costs, the requirement of deep sedation, and the requirement for endoscopists to possess expertise in EUS-guided tissue sampling, often necessitating extra training in EUS-LB [21]. In a study by Sundaram et al., EUS-LB proved to be a safe and effective alternative to PC-LB. It successfully diagnosed patients in over 95% of cases. While EUS-LB remains safe for those with mild fluid buildup (ascites) in the abdomen, ascites can slightly reduce the chance of getting the best possible tissue sample [22]. 

Earlier studies compared EUS-LB with needle biopsy done through the skin (PC-LB) to see how good each method is at getting tissue and how safe they are. One study by Pineda et al. found that EUS-LB could take longer samples, especially if they biopsied both sections of the liver. They also concluded that EUS-LB was at least as good as, and sometimes better than, the other biopsy methods [23]. However, it is worth noting that these investigations utilized first-generation fine-needle aspiration needles for EUS-LB rather than fine-needle biopsy needles. 

A recent meta-analysis, comprising five studies comparing outcomes of EUS-LB, PC-LB, and TJ-LB, found no disparities in biopsy adequacy or adverse events between EUS-LB and PC-LB, or between EUS-LB and TJ-LB. Additionally, the comparison between EUS-LB and PC-LB revealed no discrepancy [24]. What distinguishes our meta-analysis from this review is that all the studies included in our analysis utilized first-generation Fine-needle aspiration (FNA) needles. We specifically focused on studies where the majority of EUS-LB procedures employed newer second-generation needles to facilitate a more accurate comparison of outcomes with PC-LB. 

The findings of this meta-analysis provide valuable insights for clinical practice in hepatology and gastroenterology. Firstly, the comparable diagnostic adequacy and safety profiles between EUS-LB and PC-LB suggest that both procedures can be viable options for obtaining LBs. An EUS-LB offers improved accessibility to lesions in difficult locations like the left lobe or near vascular structures, real-time visualization, enhanced safety by avoiding peritoneal cavity traversal, detection of smaller lesions, concurrent therapeutic interventions, outpatient feasibility, and ascites avoidance. However, it requires specialized endoscopic expertise and equipment. A PC-LB is a more widely available, cost-effective option but carries higher risks of bleeding, organ injury, and limited lesion accessibility, especially in patients with ascites or obstructing lesions. For lesions accessible by both methods, EUS-LB may be preferred in patients with higher bleeding risk, ascites, or the need for concurrent interventions [3]. Conversely, PC-LB may be favored in resource-limited settings or for easily accessible lesions in low-risk patients. Ultimately, the choice should be individualized based on lesion characteristics, patient factors, institutional expertise, and available resources. This implies that clinicians have flexibility in choosing the biopsy technique based on patient factors, procedural preferences, and local resources. However, the observed superiority of PC-LB in terms of specimen quality, indicated by a higher number of complete portal tracts and longer specimen length, warrants consideration. In cases where specimen quality is critical for accurate diagnosis and staging, such as in the evaluation of liver fibrosis, PC-LB may be preferred. Clinicians should weigh the importance of specimen quality against other factors when selecting the biopsy approach. Overall, this meta-analysis underscores the importance of tailoring liver biopsy approaches to individual patient needs and clinical circumstances. By considering factors such as specimen quality, procedural preferences, and expertise, clinicians can optimize the diagnostic yield and safety of liver biopsy procedures. Further research and clinical experience are needed to refine our understanding of the comparative effectiveness and long-term outcomes associated with EUS-LB and PC-LB. 

The current meta-analysis is subject to several limitations that warrant consideration. Firstly, the inclusion of only nine studies, with merely two being RCTs, may limit the robustness of our findings. Additionally, the predominance of retrospective study designs among the included literature introduces the possibility of selection bias, potentially influencing the validity of our results. Furthermore, the inconsistent reporting of data pertaining to the number of passes with EUS-LB across studies poses challenges in conducting a comprehensive analysis. This variability in reporting hampers our ability to draw definitive conclusions regarding the optimal number of passes required for optimal diagnostic yield. Moreover, due to the inherent anatomical constraints associated with both EUS-LB and PC-LB, it remains uncertain whether one approach is inherently superior to the other for specific liver segments. Further investigation is warranted to elucidate the potential segment-specific advantages or limitations of each technique. Lastly, the geographical and institutional biases present in the included studies, with a majority originating from the USA and conducted in specialized centers, may limit the generalizability of our findings to broader patient populations and healthcare settings. Therefore, caution should be exercised when extrapolating these results to other regions or healthcare contexts. 

## Conclusions

In conclusion, this meta-analysis demonstrates that EUS-LB and PC-LB have comparable diagnostic adequacy and safety profiles, providing clinicians with flexibility in choosing the appropriate liver biopsy technique. However, PC-LB exhibits superiority in obtaining higher-quality specimens with more complete portal tracts. Clinicians should weigh the importance of specimen quality against procedural preferences and expertise when selecting the biopsy approach, tailoring it to individual patient needs and clinical circumstances. Further research is needed to refine the comparative effectiveness and long-term outcomes of these techniques.
